# Supplementary material for: Epidemiological impacts of the NHS COVID-19 app in England and Wales throughout its first year
Source: Nat Commun. 2023 Feb 22;14:858. doi: 10.1038/s41467-023-36495-z (PMC9947127; doi:10.1038/s41467-023-36495-z)
Supplement: Supplementary file 1 — Supplementary Information [file 41467_2023_36495_MOESM1_ESM.pdf]

Supplementary Materials

1. Supplementary Methods

We conducted our analysis in R[S1] with particular use of the packages data.table[S2], leaflet[S3], plotly[S4], RAthena[S5] and tidyverse[S6].

1.1 Active users and contact tracing enabled

Each day all devices with the app installed send a data packet to the central NHS server provided they are switched on and have internet access. We call the total number of these daily packets the number of "active users" whilst noting that it is a proxy for the actual number of active users since, for example, we do not know if an individual owns multiple phones, or if a phone is effectively inactive for contact tracing by being left at home.

The fluctuations in active users in Autumn 2020 were due to the following issues affecting Android devices: (a) Packets missing due to an Android battery optimisation feature. This was fixed in Version 3.12, released on 30 November 2020; (b) Duplication of packets since Version 3.10, released on 9 November 2020. This was fixed in Version 4.1, released 17 December 2020. Wymant & Ferretti et al. estimated the number of users in this period to be 16.5 million[S7] but this number is likely to be in excess of the actual number of app users, being affected by the issues outlined above.

We assign each user to their self-declared Lower Tier Local Authority (LTLA) or, when this is unavailable, we assign them to the LTLA to which most of their self-declared postcode district (the first half of the postcode) belongs. We note that, to further preserve anonymity, where postcode districts have a population of less than 5000, data is automatically amalgamated with nearby districts using a lookup from the ONS. This is intended to preserve the LTLA mapping but minor discrepancies are possible. The app does not collect any demographic data about users.

As described in the public app data notes[S8], the number of users with contact tracing enabled is based on the estimated number of users with the app installed where the app is deemed 'usable' and 'contact-traceable'. The app is deemed usable if the version of the app is supported and onboarding has been completed. It is deemed contact-traceable if, in addition to being usable, Bluetooth is enabled and the user is able to receive exposure notifications. The app is deemed not usable or not contact-traceable if the device is running Android OS versions 6 to 10 and location sharing is disabled.

1.2 Exposure notifications

NHS COVID-19 app exposure notifications work as follows. The overarching aim is to implement automated contact tracing in a way that preserves privacy: individuals are notified of past contacts with infected cases, but no central graph of who contacted whom is ever

constructed or stored.[S9] Each installation of the app on a device has a unique key that regularly changes. When two individuals with the app, A and B say, are in close proximity, their apps each record the key of the other and retain it in memory for 14 days. When A reports a positive test through the app and consents to 'key sharing', their app shares with the central NHS server the list of keys that it has recently used to identify itself (not the list of keys from other devices it has encountered, to preserve privacy). This is recorded as one test-positive event. When B's app next checks the list of keys on the NHS server for positive individuals, it recognises one of A's keys as one stored in its memory, i.e. it recognises A as a test-positive contact of B.

Other information about the contact event is analysed on B's device: the contact event is given a score based on duration, proximity, and time relative to self-declared onset of symptoms (with contacts closer to time of symptom onset more likely to result in transmission). If the score is above a threshold, the user B is notified of a "risky" close contact. This event is recorded as an exposure notification. The threshold score and risk-calculating algorithm have undergone changes since app launch, but the threshold has been constant since 1 January 2021. For consistency with public health policy, the risk threshold is approximately equivalent to the risk of an exposure to a standard infectious individual at a distance of 2 metres for 15 minutes.

For phones using app versions earlier than 4·1 (which was released on 17 December 2020) we approximate the number of exposure notifications received using the following conditions on app data fields:
0 < hasHadRiskyContactBackgroundTick < runningNormallyBackgroundTick
AND runningNormallyBackgroundTick > 0
AND isIsolatingBackgroundTick > 0.
(The first condition means the data indicate that the app spent part of the current day, but not all of the day, notified of recent risky contact; this should occur on the first day of isolation and not the last. The second and third conditions mean the data indicate that the app is running normally and the individual has been advised to isolate.) This approximation is likely to underestimate the number of exposure notifications received. For devices which upgraded to Version 4.1 or higher (most phones upgrade within a day or two of the release) we are able to use these fields alongside the confirmatory isIsolatingForHadRiskyContactBackgroundTick > 0 for a more accurate timeseries of total daily notifications.

1.3 Key sharing

As explained above, when a user records a positive test result in the app they are given the option to alert their recent contacts. If they consent, their recent 'keys' are shared with the central NHS server. The proportion of users consenting to key sharing varies over time (Figure S1). After the release of Version 4.32 we are able to track this using the successfullySharedExposureKeys data field. The measure increased steadily through June and July, reaching steady values of 50-55% in August and September. We do not know the reason for this increase - it is likely that some of it can be attributed to the gradual process of users upgrading to the latest app version, but the majority of users typically upgrade within 1-3 days of

a version release. In the absence of data before 11 June 2021 we suggest a fixed estimate of 40%, but we do not use this measure in our analysis...

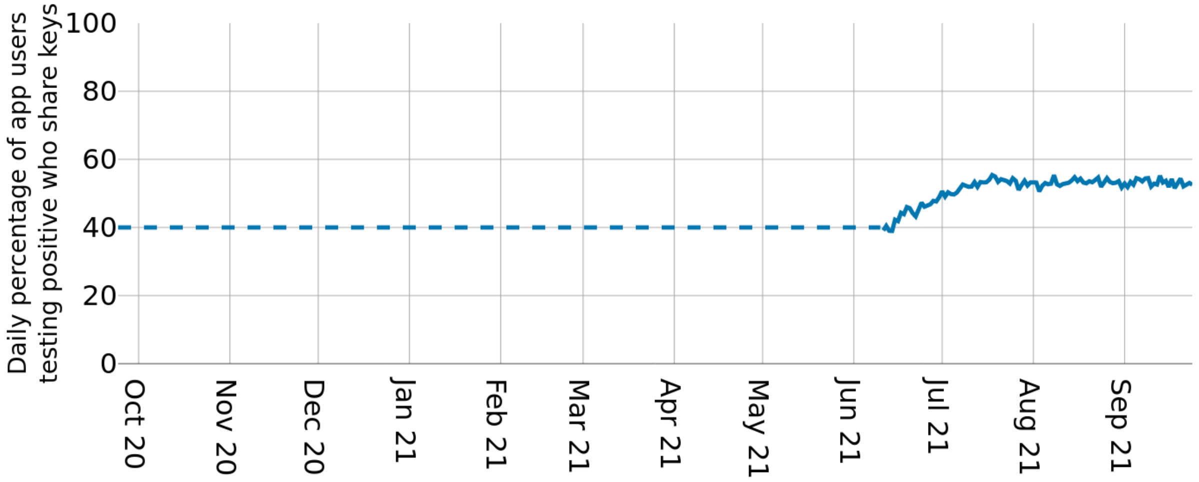

Figure S1. The percentage of test-positive app users who consent to alert their recent contacts ie. who consent to key sharing.

1.4 Manual tracing

For the number of manually traced contacts per case we use “NHS Test and Trace statistics 28 May 2020 to 6 April 2022: data tables” from the weekly statistics webpage.[S10] We take the number of “close contacts reached and asked to isolate” (Table 13, row 5) and divide by the “number of people reached and asked to provide details of recent close contacts” (Table 10, row 5). To the best of our knowledge, “asked to isolate” incorporates those asked to isolate and those advised to test, following the 16 August 2021 advice change (see Timeline of Events below for more details). For the breakdown of contacts by household status we use for the respective numerators “Total number of contacts identified not managed by local [Health Protection Teams] HPTs who were household contacts: close contacts reached and asked to self-isolate (Table 14, row 10) and “Total number of contacts identified not managed by local HPTs who were non-household contacts: close contacts reached and asked to self-isolate (Table 14, row 15). Household status for contacts managed by local HPTs is not available but such contacts comprise between 6.0% and 0.2% of the total during the period of study (Table 10, row 15).

1.5 TPAEN

The proportion TPAEN is the proportion of app users who report a positive test through the app in a time window starting with their notification of a risky exposure and ending 14 days after their recommended isolation period. We previously[S7] referred to this statistic as the Secondary Attack Rate (SAR). However, SAR is a generic term often used in epidemiology to refer instead to the fraction of contacts who are truly infected; the fraction of contacts who are detected as cases

differs from this depending on case ascertainment and related factors such as availability of testing and propensity to get tested. For this reason, we prefer to use a term that refers explicitly to the only outcome we can observe: whether a positive test is reported through the app in the aforementioned window, and not whether the contact is truly infected.

To calculate the proportion TPAEN we model daily numbers of notifications and positive tests among those recently notified as negative-binomially distributed about their expected values. Our likelihood is

$$N_t \sim \text{NegBin}(\hat{N}_t,\ \phi)$$
$$P_t \sim \text{NegBin}(\hat{P}_t,\ \phi)$$

where

$$\hat{N}_t = \exp(\tilde{n}t + \sum_b B_{t,b} n_b)$$
$$\hat{F}_t = \exp(\tilde{f}t + \sum_b B_{t,b} f_b)$$
$$\hat{P}_t = \sum_{t' < t} \hat{N}_{t'} \hat{F}_{t'} P_{n-to-p}(t - t')$$

$$n_b = \hat{n} n'_b$$
$$f_b = \hat{f} f'_b$$

Discrete time t is the number of days since 1 January 2021.
N_t is the observed number of notifications at time t.
P_t is the observed number of positive tests reported through the app from those 'recently notified' (in their isolation or in the 14 days after), at time t.
$\hat{N}_t$ is the expected number of notifications at time t.
$\hat{P}_t$ is the expected number of positive tests reported through the app from those 'recently notified', at time t.
ϕ is the negative binomial overdispersion parameter, defined such that NegBin(μ, ϕ) has mean μ and variance $\mu + \mu^2 / \phi$, with the limit $\phi \rightarrow \infty$ recovering a Poisson distribution with no overdispersion.
$\tilde{n}$ allows for an overall trend in notifications over time, parameterising a tendency toward exponential growth (or decline).

$B_{t,b}$ is the contribution of the bth basis function to the spline at time t, defined such that

$\sum_b B_{t,b} = 1, \forall t.$

n_b is the logarithmic contribution to the number of notifications from the bth component of the spline.

$\hat{F}_t$ is the expected fraction of notifications at time t that will go on to report a positive test through the app in the near future (while still 'recently notified'), i.e. this is the proportion TPAEN at time t.

$\tilde{f}$ allows for an overall trend in the proportion TPAEN over time, parameterising a tendency toward exponential growth (or decline).

f_b is the logarithmic contribution to the proportion TPAEN from the bth component of the spline.

$P_{n-to-p}(t'')$ is the probability that an app user who is notified will report a positive test while still 'recently notified' a time t'' after their notification, conditional on their reporting a positive test while still 'recently notified' at some time, i.e. $\sum_{t''} P_{n-to-p}(t'') = 1.$

$\hat{n}$ defines the scale of the variability in logarithmic contributions to the number of notifications between the different knots in the spline.

n'_b defines the (scale-standardised) variation in n for the bth component of the spline.

$\hat{f}$ defines the scale of the variability in logarithmic contributions to the proportion TPAEN between the different knots in the spline.

f'_b defines the (scale-standardised) variation in f for the bth component of the spline.

We ignored the first month of observations when calculating the likelihood: each day's contribution to the likelihood depends on a sum over the past, so we allowed time for the observations to settle. In a minor violation of the Bayesian paradigm, two high-level features of the data were used to set weakly informative priors on the overall scale of notifications and the proportion TPAEN. Namely, the mean daily number of observed notifications $\langle N \rangle$ and the mean daily number of observed positives among those recently notified $\langle P \rangle$. Our priors were

$$\begin{aligned} \phi^{-1} &\sim \text{Half-Cauchy}(0, 1) \\ \hat{n} &\sim \text{Cauchy}(0, \text{abs}(\log(5\langle N \rangle))) \\ \hat{s} &\sim \text{Cauchy}(0, \text{abs}(\log(5\langle P \rangle / \langle N \rangle))) \\ n'_b &\sim \text{Normal}(0, 1) \\ f'_b &\sim \text{Normal}(0, 1) \end{aligned}$$

The model above was implemented in Stan[S11], running the chains for 2000 iterations (with 50% burn in) with target average acceptance probability of 0.95 and other parameters set to their default values. These parameters result in a good mixing for this model (maximum $\widehat{R}$ = 1.022, minimum n_{eff} > 300).

1.6 Prevalence

We use prevalence estimates from the ONS Coronavirus infection survey[S12], using Table 1j: "Non-overlapping 14-day weighted estimates of the percentage of the population testing positive for COVID-19 by age/school year, England". Equivalent data for Wales (by age range) was not available. We then use ONS population structure from mid 2020[S13] to construct a fortnightly weighted average prevalence across over 16s for England. We then plot the fortnightly centred mean proportion TPAEN from app data divided by the fortnightly mean prevalence across over 16s for England.

1.7 Relative incidence

To demonstrate the relative incidence we plot daily measures of the following: the proportion
"reporting a positive test result and recently notified" / "recently notified" * 100,000
alongside the proportion
"reporting a positive test result and not recently notified" / "not recently notified" * 100,000.

1.8 Assignment of positive tests and notifications to variants

In order to assign positive tests and notifications to the pre-Alpha wave or to the Alpha wave, we weight the positive tests and exposure notifications over the Autumn-Winter 2020 wave with the relative fraction of Alpha exponentially increasing at a daily rate of 0.08 (median value among regressions based on S-gene target failures[S14]) so that the Alpha variant comprises 50% of cases by 21 December 2020 (since S-gene target failures became dominant in England after week 50 of 2020). For the Delta variant such a weighting is not needed, since the variant started with a number of introductions at a time when the number of cases was very low. For simplicity, we consider the Delta wave to start on 17 May 2021, since the majority of the sequences from the UK after that date belong to this variant. The weights for the different waves are reported in Figure S2. We split the data into these three "variant waves" so that, in our estimates of cases averted, we do not allow counterfactual transmission chains to continue from one wave into the next. That is, whenever we count an averted pre-Alpha case, we do not permit a calculation which would implicitly assume that this individual could have transmitted an onward Alpha infection (and similarly from Alpha to Delta).

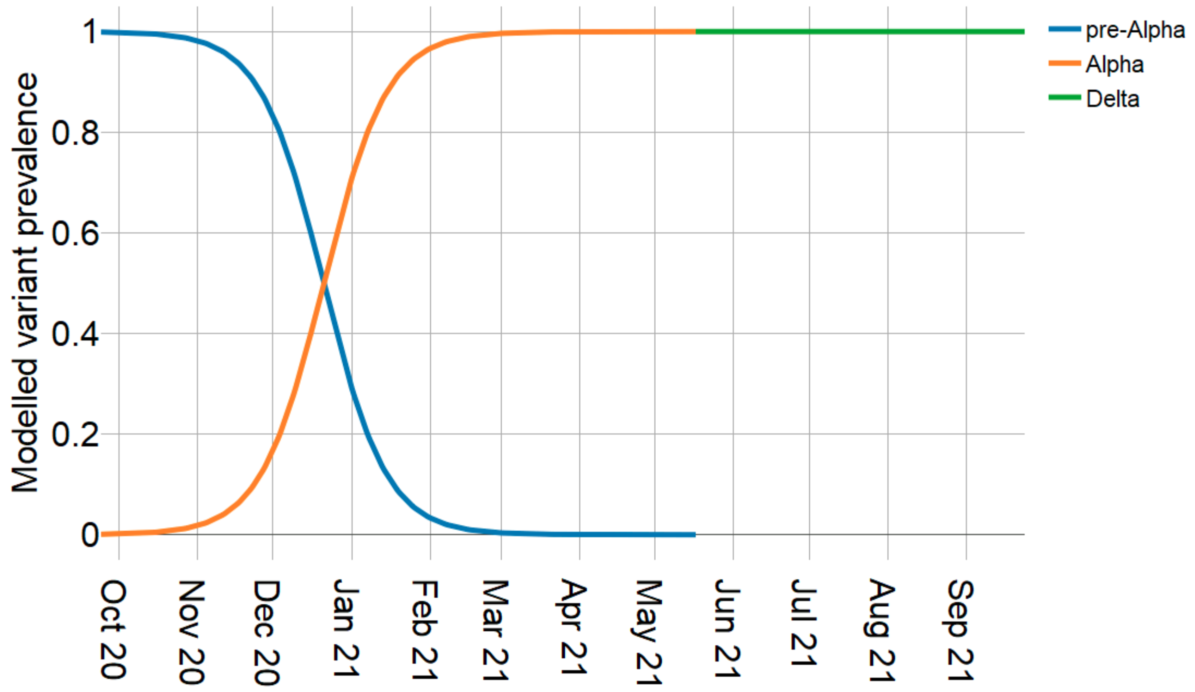

Figure S2. Simple model of variant prevalence.

1.9 Estimated timings and compliance

We know that there are variations in the occurrence, order and timings of the following possible events which may or may not occur within an individual's SARS-CoV-2 infection:

(a) detection of symptoms
(b) booking of a test
(c) taking a test
(d) receiving a test result
(e) supplying details of recent contacts to Test and Trace
(f) consenting to contact tracing via the NHS COVID-19 app
(g) receiving contact tracing alert(s) from Test and Trace
(h) receiving a contact tracing alert from the NHS COVID-19 app

These may vary between individuals and over calendar time.

We were unable to find data which is detailed enough to give a clear picture of the relative occurrences and timings of such events. Data from UKHSA[S10] provides some insight into relevant timings (Figure S3) when viewed in the context that app notifications are received within 4 hours of the index case receiving or registering their positive test in the app and consenting to contact tracing, and the contact having their phone switched on. This lends support to the assumption that app notifications can arrive faster than non-app contact tracing alerts, and before an individual receives a positive test result for a suspected case (but not necessarily if the individual is testing routinely and often). We use this later to inform the general

shape of Figure S4, where it seems reasonable to assume that the green shaded area has some positive value.

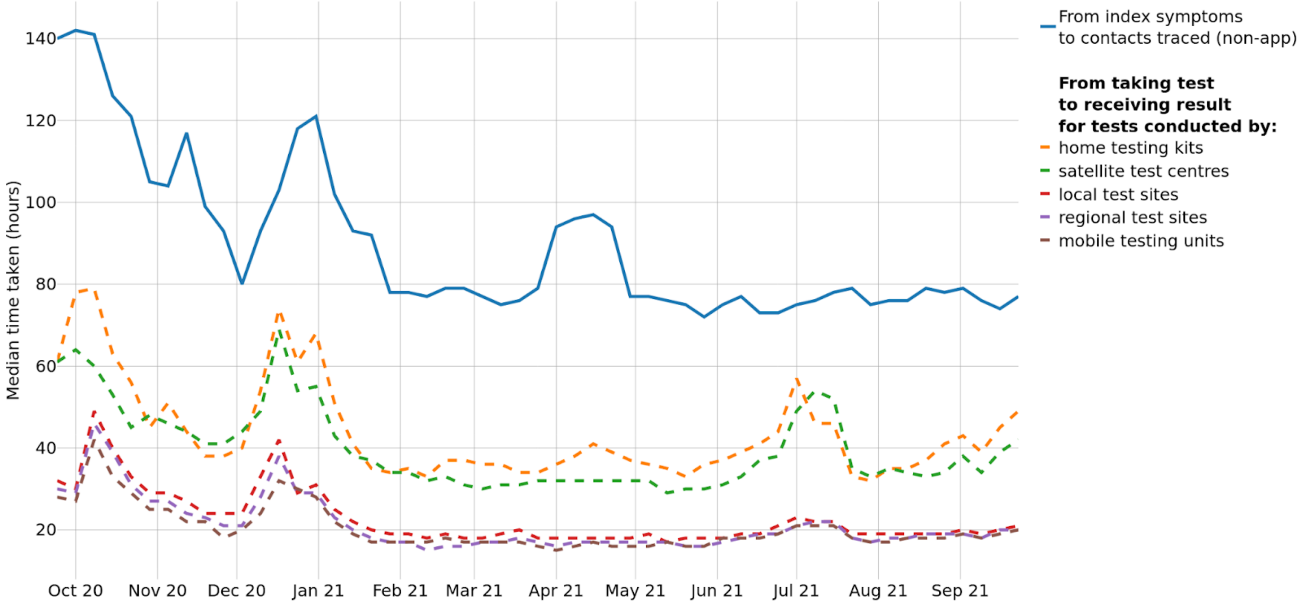

Figure S3. Timings between events as published by UKHSA.[18]

We compare the empirical distribution for the time taken from app notification to positive test to the generation time distribution from Ferretti et al.[S15] to estimate the proportion of the infectious period remaining for the notified individual over the days following an app notification. These estimates are partially informed by data[S7,S16] but could be made more precise if further survey data becomes available.

1.10 Sensitivity analysis and modelling the interaction with other PHSMs

Anonymised app data presents challenges for drawing comparisons with and understanding interactions with other Public Health and Social Measures (PHSMs) designed to reduce SARS-CoV-2. Table S1 lists the data we would need to perform a thorough evaluation of the app in the context of other measures. We list the data that is available or not, to the best of our knowledge, showing that we have insufficient data for a definitive result. Instead we use a simple model which could be developed as more data becomes available, and we present extremal results here in a brief sensitivity analysis to demonstrate a plausible range of measures of the app’s impact.

Event during infection	Timing in the course of infection	Behavioural change due to event	Intersection with app users
Manual tracing	Partial data for England only as shown in Figure S3[S10]	Partial data from ONS, England only[S16]	Not available
Word of mouth tracing	Not available	Not available	Not available
Routine LFD testing	Availability varied over calendar time. Data is available on the total number of tests taken and reported per day. We are unaware of data on the frequency of routine testing and its relationship to the onset of infection.	Partial data from ONS, England only[S16]	Not available
Symptoms	Timing of the onset of symptoms relative to the onset of infection is given in Ferretti et al.[S15]	Partial data from ONS, England only[S16]	The app includes a symptom checker but this is an under-used feature and does not collect enough data to understand the timing of the onset of symptoms for a user.
Positive PCR result	Partial data for England only as shown in Figure S3[S10]	Partial data from ONS, England only[S16]	Not available

Table S1. Data required for an exact analysis, and its availability to the best of our knowledge.

First, we consider the extreme where other PHSMs were negligible: that the app was the only means by which individuals could discover their infected status, and an app notification was the only event which would cause a user to reduce their “usual” numbers of risky contacts. Although this is clearly unrealistic, it offers a glimpse as to the potential for an app to reduce transmission with less reliance on other PHSMs. Using the same assumptions as in the main text about compliance and behavioural change following an app notification, this leads to an estimate of 1.4 million cases averted by the app (780,000 to 2,000,000 with lower and upper levels of risky contact reduction respectively).

It is more realistic and informative to consider the app in the presence of other PHSMs and attempt to estimate the particular impact of the app against a counterfactual where the app is

not present but everything else is identical. The mathematical model we use for this is as follows. Let t be the time since an individual was infected. Let $P_a(t)$ be the probability that the individual was app-notified by time t. Let ω(t) be the probability that an infected individual has become aware of their infection status by other means (for example by word of mouth tracing, manual tracing, onset of symptoms, or LFD or PCR testing) by time t. (See Figure S5 and later text for full details of how we model ω(t).) We model app notification and discovery of infection status by other means as independent, such that the probability of being both app-notified and aware by other means by time t is $P_a(t)\,\omega(t)$. Let $\beta_0(t)$ be infectiousness (the instantaneous hazard for transmission) at time t in the absence of any interventions, with a relative infectiousness (less than 1) of r_a upon being app-notified and of r upon becoming infection-aware by other means. Let β(t) be infectiousness in the presence of interventions: this equals $\beta_0(t)$ weighted by the probability of different interventions and the reduction in infectiousness that they cause. Conservatively assuming that there is no additional benefit to app notification if one is already aware of being infected by other means—i.e. that the relative infectiousness of such individuals is r, the same as those only aware by other means—the overall infectiousness is thus

$$\beta(t) \ = \ \beta_0(t)\,(\omega(t) r \ + \ (1 - \ \omega(t))(P_a(t) r_a \ + \ 1 - P_a(t)).$$

To obtain the overall infectiousness in the absence of the app while keeping other PHSMs in place, we need only set $P_a(t)$ to zero in the above expression. The reduction in infectiousness due to the app is then obtained by subtracting infectiousness with the app from infectiousness without the app:

$$\beta_{app-averted}(t) \ = \ \beta_0(t)\,(1 - \ \omega(t)) P_a(t)(1 - r_a).$$

The reproduction number is given by the integral of infectiousness over the course of infection,[S17] and so the reduction in the reproduction number due to the app is given by the integral of $\beta_{app\text{-}averted}(t)$ over t.

Recall that our calculation of cases averted by the app proceeds by multiplying together (i) the number of notifications, (ii) the proportion of those notified who are infected, (iii) the fraction of the infectious period which occurs between an individual receiving an app notification and before discovering their infected status via another means, (iv) the individual's fractional reduction in risky contacts i.e. in their reproduction number as a result of an app notification, and (v) the expected size of the onward transmission chain which would have originated with the contact had they not been notified. In the model described above, β(t) referred to the infectiousness of an average infected individual; for component (iv) in this product, we are conditioning on the individual receiving an app notification at some point, i.e. we want the reduction in the reproduction number specifically for app-notified infected individuals. This means only that $P_a(t)$ should be scaled such that it asymptotes to 1 at large t, not to the probability that the average infected individual eventually receives an app notification. $P_a(t)$ is thus the cumulative distribution function of the probability density function for the time from becoming infected to being app-notified, conditional on being app-notified at some point..

Figure S4 illustrates the idea by showing three curves of infectiousness over the course of an infection. In blue is the infectiousness for an individual in the absence of any intervention. In orange is the infectiousness conditional on an individual never being app notified, marginalising over whether they are notified by other means and if so when (i.e. integrating over the timing distribution implied by $\omega(t)$). In green is the infectiousness of an individual conditional on their being app notified at t = 5 days, marginalising over whether they are notified by other means and if so when. The green shaded area between the green and orange curves is the average reduction in the reproduction number R caused by an app notification at t = 5 days relative to a counterfactual with no app notification but all other PHSMs in place. Marginalising over whether an individual is app notified and if so when, we arrive at the total reduction in R due to the app relative to the counterfactual.

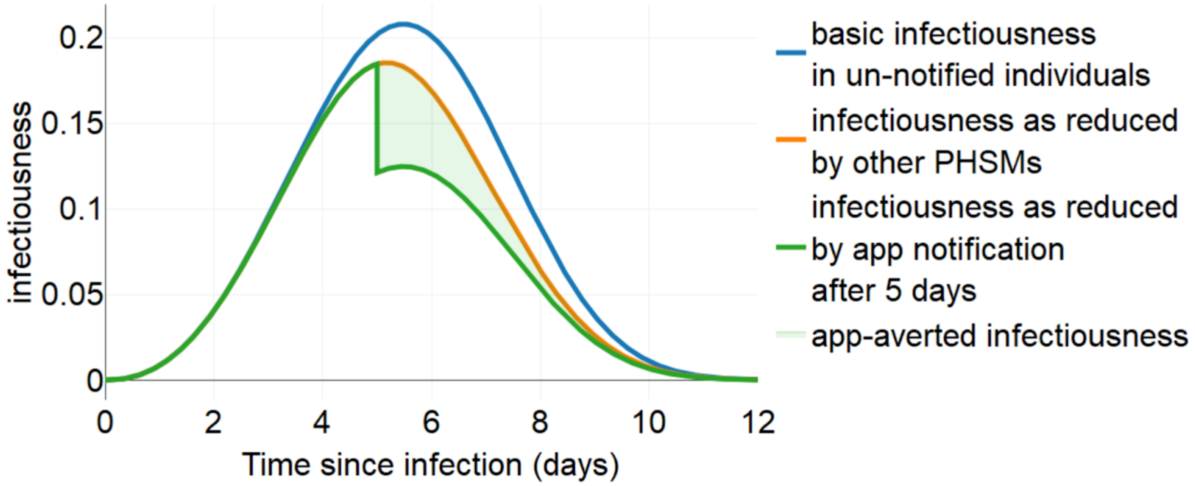

Figure S4. The infectiousness curve in the absence of PHSMs (blue), and its modifications as reduced by various PHSMs.

For each date we calculate the reduction in reproduction number as a result of app notification (the integral of $\beta_{\text{app-averted}}(t)$ over t) for individuals who are app-notified on that date. This varied over calendar time due to the fact that the delay from exposure to notification varied. We estimate this delay from additional app data, conditioning on the notified individual later testing positive, and found that it had a mean of 4 days, range 2.1 to 7.4 days, after a one-week burn-in period from the app's launch.

The fraction ω of app-notified infected individuals who are also notified another way is time-dependent, both in calendar time and during an individual infection (Figure S5). We suppose that it grows during the course of infection, from 0 at the moment of infection to Ω by day 12, when the Weibull-distributed infectiousness becomes negligible.[S15]

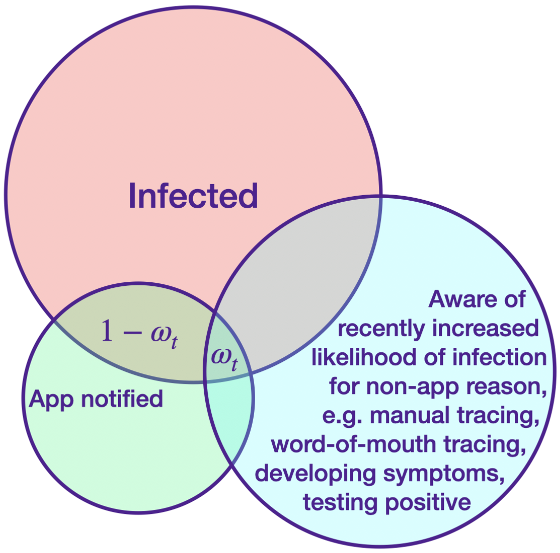

Figure S5. The fraction ω_t of app-notified infected individuals who are also notified another way varies over the course of infection.

We model this simply with a logistic distribution:

$$\omega_t = \Omega / (exp(- k(t - m)) + 1)$$

For conservative estimates of the app's effect, we chose $\Omega = \omega_{12} = 1$ for the Delta wave when LFDs were readily available, and $\Omega = 0.8$ for the pre-Alpha and Alpha waves. It is possible that these estimates should be considerably lower because ONS infection survey estimates have typically shown the number of infections to be around 2 or 3 times the number of positive tests reported. Setting $\Omega = 0$ gives the extremal calculation above where app notifications are the only means by which individuals discover they are infected and change their behaviour. We note that the existence of LFDs later in the period of study may also change the shape of the infectiousness curve itself but we did not have sufficient data to estimate this. We chose parameter values $k = 1$/day and $m = 6$ days, motivated by 6 days being the median of the generation time. Again, the analysis would be improved if data becomes available to inform more accurate choices for these parameters.

We assume that individuals in the intersection ω (app notified and also notified another way) reduce their risky contacts by a factor $0 \leq r \leq 1$ compared to general background contact rates at the time. For our central estimates we use r = 0.4, i.e. people on average retain 60% of the contacts they would have had the previous week. Our model allows for this to change with each wave but we suppose it to be constant for the following reasons. In times of strong restrictions an average individual's typical daily risky contacts are likely to be predominantly within-household, and so may continue even once an infection is discovered. On the other hand, during the Delta wave there were fewer restrictions but, with a background of high

vaccination coverage, popular attitudes and survey data indicate probable decreasing compliance with guidelines.[S18]

Finally, to estimate the size of the chain of transmissions resulting from a single case (factor (v) in the calculation), we follow the method of Wymant & Ferretti[S7] of attributing to each averted case on day *t* a fraction 1 / (*rolling_7-day_average_cases(t) * generation_time)* of all future cases for that wave. We perform the calculation separately for each LTLA before summing to the national level and, as discussed in Supplementary Section 1.8, we restart the calculation for each of the three variant waves.

We present a sensitivity analysis varying Ω and r in Figure S6. For simplicity we consider r constant across waves, and $\Omega_{\text{pre-Alpha}} = \Omega_{\text{Alpha}} = \Omega_{\text{Delta}} - 0.2$ to account for the presence of LFDs (which provide a further means for detecting asymptomatic infections).

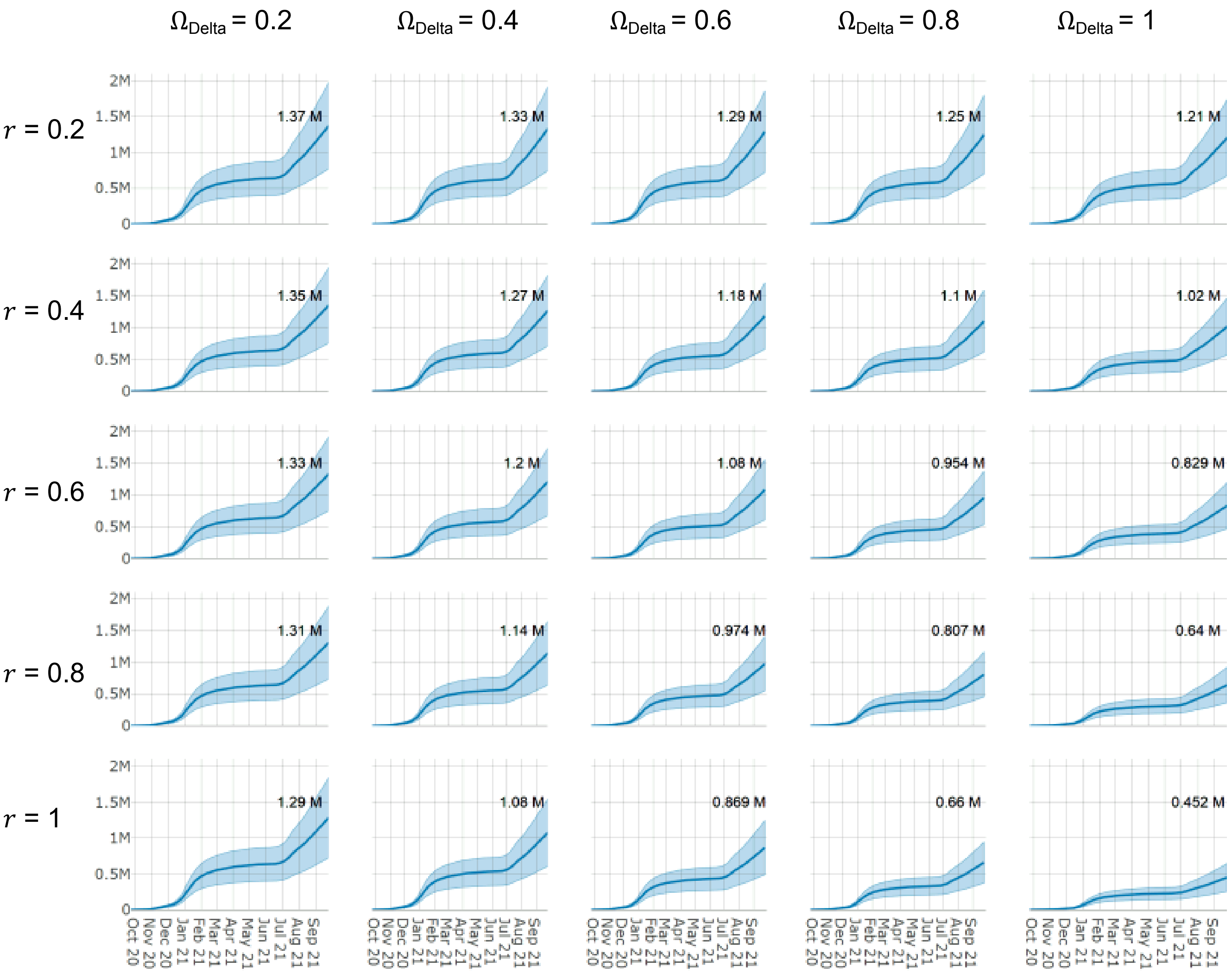

Figure S6. Sensitivity analysis for the number of cases averted. Top left: only 20% of infected individuals ever find out via other PHSMs that they're infected, and they only reduce their risky contacts by 20% when they do. Bottom right: 100% of infected individuals discover via other PHSMs that they are infected, and reduce their risky contacts by 100% (perfect isolation) when they do. Shading indicates the range of outcomes between upper and lower plausible estimates of an individual's reduction in risky contacts as a result of receiving an app notification.

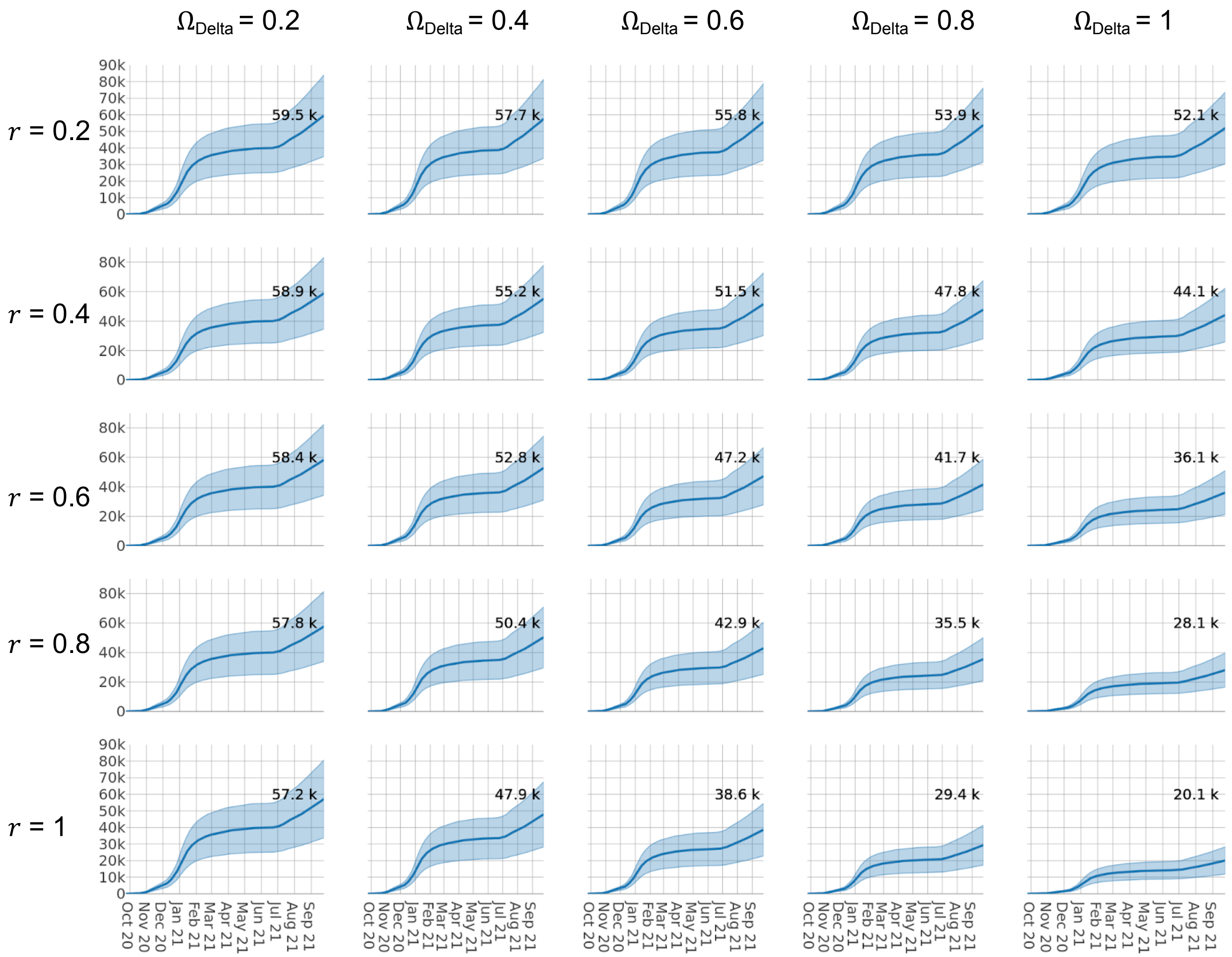

Figure S7. Sensitivity analysis for the number of hospital cases averted. Top left: only 20% of infected individuals ever find out via other PHSMs that they're infected, and they only reduce their risky contacts by 20% when they do. Bottom right: 100% of infected individuals discover via other PHSMs that they are infected, and reduce their risky contacts by 100% (perfect isolation) when they do. Shading indicates the range of outcomes between upper and lower plausible estimates of an individual's reduction in risky contacts as a result of receiving an app notification.

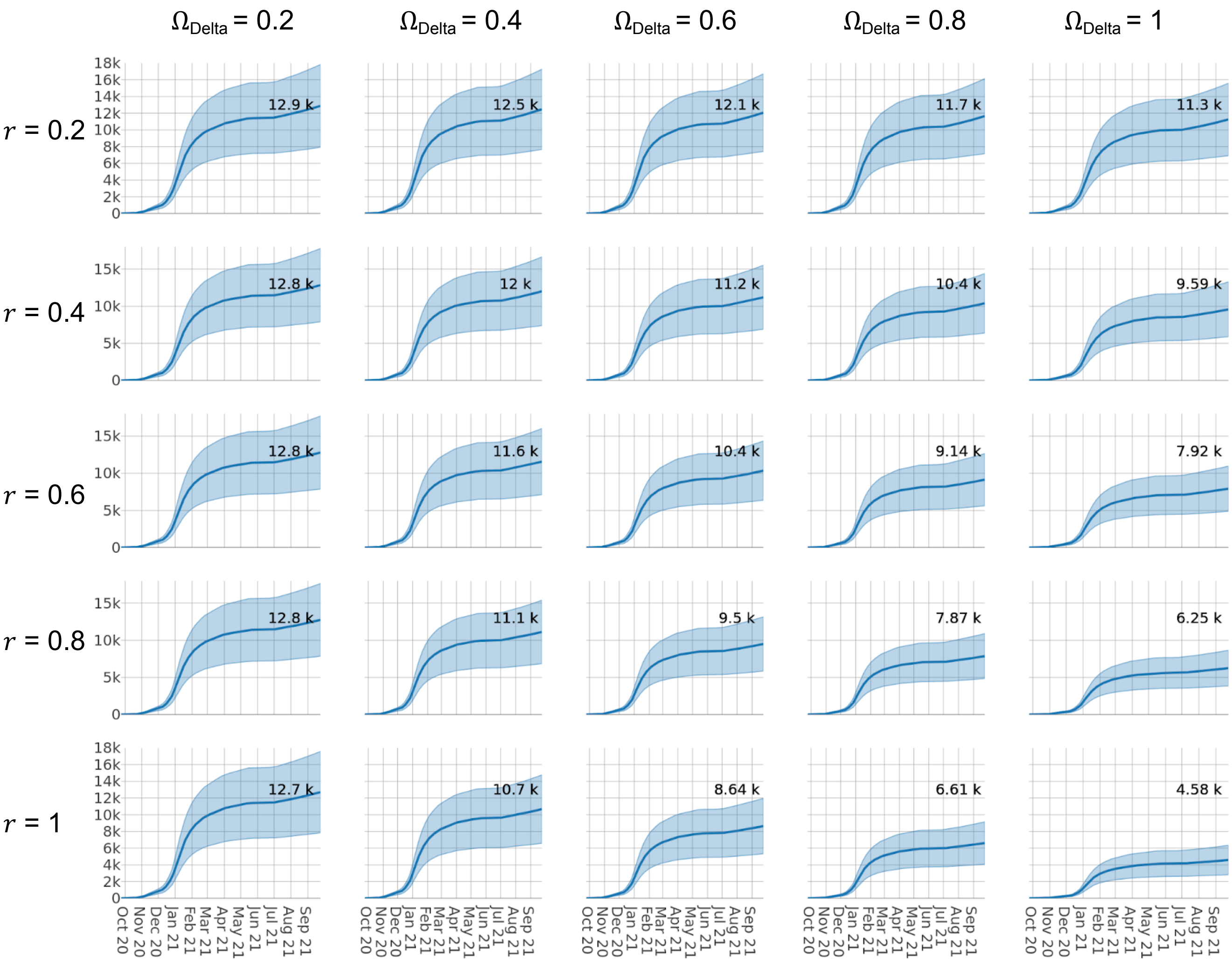

Figure S8. Sensitivity analysis for the number of deaths averted. Top left: only 20% of infected individuals ever find out via other PHSMs that they're infected, and they only reduce their risky contacts by 20% when they do. Bottom right: 100% of infected individuals discover via other PHSMs that they are infected, and reduce their risky contacts by 100% (perfect isolation) when they do. Shading indicates the range of outcomes between upper and lower plausible estimates of an individual's reduction in risky contacts as a result of receiving an app notification.

2. Timeline of national and app events

We present a brief timeline of events which are significant for interpretation of app data, some of which are marked on relevant plots. Since 96% of app users recorded an English postcode district for their residence (4% a Welsh one) we highlight changes in English epidemic restrictions below. We note that behavioural changes sometimes occur in anticipation of a policy change.

Roadmap Step 1, 8 and 29 March 2021, and Step 2, 12 April 2021
App use increased slightly following Step 1 and again following Step 2 of the roadmap out of lockdown (Figure 1).[S19]

Roadmap Step 3, 17 May 2021
App use increased rapidly after Step 3 of the roadmap[S19] (Figure 1), likely driven by the QR code venue check-in feature.

Roadmap Step 4, 19 July 2021 and media coverage
The use of QR code check-in declined following the changes around Step 4 of the roadmap.[S19] The app received extensive media coverage in July focused on the number of requests for self-isolation in what became known as the ‘pingdemic’. There was a high rate of app deletions from mid-July to mid-August; deletions subsequently slowed, possibly owing to the positive advertising campaign.

Logic change, 2 August 2021
On 2 August 2021 the contact tracing logic of the app was changed[S20] so that it only notifies close contacts from the two days before an asymptomatic index case tests positive, rather than the previous five days. This was expected to somewhat reduce the number of app notifications per index case but it is hard to discern a clear impact from the timeseries (Figure 4b). Note that, contrary to some news reports, the logic for symptomatic index cases was not changed and there was no change to the risk threshold of the app.

Advice change, 16 August 2021
From 16 August 2021 until the end of our study period on 24 September 2021, in line with national policy, users who were contact traced were no longer advised to self-isolate but were advised to take a PCR test if they self-declared that they: were fully vaccinated, or were aged under 18 years and 6 months, or were medically unable to be vaccinated, or had been in trials for non-MHRA approved vaccines.[S21] It is possible that the advice to test drove more positive index case-finding, or at least helped detect infected individuals earlier in their infectious period.

Supplementary References

S1. R Core Team (2021). R: A language and environment for statistical computing. R Foundation for Statistical Computing, Vienna, Austria. Available from: https://www.R-project.org/

S2. Dowle M and Srinivasan A (2021). data.table: Extension of `data.frame`. R package version 1.14.2. Available from: https://CRAN.R-project.org/package=data.table

S3. Cheng J, Karambelkar B, Xie Y (2022). leaflet: Create Interactive Web Maps with the JavaScript 'Leaflet' Library. R package version 2.1.1. Available from: https://CRAN.R-project.org/package=leaflet

S4. Sievert C. Interactive Web-Based Data Visualization with R, plotly, and shiny. Chapman and Hall/CRC Florida, 2020. R package version 4.10.0. Available from: https://cran.r-project.org/web/packages/plotly/index.html

S5. Jones D (2022). RAthena: Connect to ‘AWS Athena’ using ‘Boto3’ (‘DBI’ Interface). R package version 2.6.1. Available from https://github.com/DyfanJones/RAthena

S6. Wickham H, Girlich M (2022). tidyr: Tidy Messy Data. https://tidyr.tidyverse.org, https://github.com/tidyverse/tidyr

S7. Wymant C, Ferretti L, Tsallis D, Charalambides M, Abeler-Dörner L, Bonsall D, et al. The epidemiological impact of the NHS COVID-19 app. Nature. 2021 May;594:408–412.

S8. NHS COVID-19 app statistics: notes on the data [Internet]. UK Health Security Agency 2022; [updated 2022 Nov 17; cited 2022 Nov 18]. Available from: https://www.gov.uk/government/publications/nhs-covid-19-app-statistics/nhs-covid-19-app-statistics-notes-on-the-data

S9. Apple Google. Privacy-Preserving Contact Tracing [Internet]. 2020 [last accessed 2021 Aug 17]. Available from: https://covid19.apple.com/contacttracing

S10. UK Health Security Agency. Weekly statistics for NHS Test and Trace (England) [Internet]. UK Health Security Agency 2022 [updated 2022 Apr 14; cited 2022 Aug 17]. Available from: https://www.gov.uk/government/publications/weekly-statistics-for-nhs-test-and-trace-england-31-march-to-6-april-2022

S11. Carpenter B, Gelman A, Hoffman MD, Lee D, Goodrich B, Betancourt M et al. Stan: A Probabilistic Programming Language. Journal of Statistical Software. 2017;76(1):1–32.

S12. Office for National Statistics. Coronavirus (COVID-19) Infection Survey: England [Internet]. 2022 [updated 2022 Aug 5; cited 2022 Aug 17]. Available from: https://www.ons.gov.uk/peoplepopulationandcommunity/healthandsocialcare/conditionsanddiseases/datasets/coronaviruscovid19infectionsurveydata

S13. Office for National Statistics. Estimates of the population for the UK, England and Wales, Scotland and Northern Ireland. Mid-2020 edition [Internet]. 2021 [updated 2021 Jun 25; cited 2022 Aug 17]. Available from: https://www.ons.gov.uk/peoplepopulationandcommunity/populationandmigration/populationestimates/datasets/populationestimatesforukenglandandwalesscotlandandnorthernireland

S14. Davies NG, Abbott S, Barnard RC, Jarvis CI, Kucharski AJ, Munday JD et al. Estimated transmissibility and impact of SARS-CoV-2 lineage B.1.1.7 in England. Science. 2021 Mar;372(6538):eabg3055.

S15. Ferretti L, Ledda A, Wymant C, Zhao L, Ledda V, Abeler-Dörner L et al. The timing of COVID-19 transmission. medRxiv preprint. 2020. Available from: https://doi.org/10.1101/2020.09.04.20188516

S16. Office for National Statistics. Coronavirus and self-isolation after being in contact with a positive case in England [Internet]. 2021 [updated 2021 Sep 8; last accessed 2021 Aug 17]. Available from: https://www.ons.gov.uk/peoplepopulationandcommunity/healthandsocialcare/conditionsanddiseases/datasets/coronavirusandselfisolationafterbeingincontactwithapositivecaseinengland

S17. Wallinga J and Lipsitch M. How generation intervals shape the relationship between growth rates and reproductive numbers. Proc. Biol Sci. 2007;274:599-604.

S18. Fancourt D, Bu F, Mak HW, Paul E, Steptoe A. COVID-19 Social Study, Results Release 34. Available from: https://www.covidsocialstudy.org/_files/ugd/3d9db5_e381fd8742d247bdb6f5ab8eda4b9cc5.pdf

S19. UK Cabinet Office. COVID-19 Response - Spring 2021 (Summary) [Internet]. 2021 [updated 2021 Feb 22; last accessed 2023 Jan 26]. Available from: https://www.gov.uk/government/publications/covid-19-response-spring-2021/covid-19-response-spring-2021-summary

S20. Department of Health and Social Care. Press release: NHS COVID-19 app updated to notify fewer contacts to isolate [Internet]. 2021 [updated 2021 Aug 2; last accessed 2023 Jan 26]. Available from: https://www.gov.uk/government/news/nhs-covid-19-app-updated-to-notify-fewer-contacts-to-isolate

S21. Department of Health and Social Care. Press release: Self-isolation removed for double-jabbed close contacts from 16 August [Internet]. 2021 [updated 2021 Aug 11; last accessed 2023 Jan 26]. Available from: https://www.gov.uk/government/news/self-isolation-removed-for-double-jabbed-close-contacts-from-16-august
